# Analysis of Quasi-Zenith Satellite System Signal Acquisition and Multiplexing Characteristics in China Area

**DOI:** 10.3390/s20061547

**Published:** 2020-03-11

**Authors:** Hongjun Ye, Xiaojun Jing, Liang Liu, Maolei Wang, Shuo Hao, Xingkang Lang, Baoguo Yu

**Affiliations:** 1 School of Information and Communication Engineering, Beijing University of Posts and Telecommunications, Beijing 100876, China; jxiaojun@bupt.edu.cn; 2 State Key Laboratory of Satellite Navigation System and Equipment Technology, Shijiazhuang 050081, China; 15097335662@163.com (L.L.); alizhuomian@163.com (S.H.); 17718570869@163.com (X.L.); yubg@sina.cn (B.Y.); 3 Beijing Satellite Navigation Center, Beijing 100876, China; 15246783497@163.com

**Keywords:** QZSS, navigation signal, identification of multiplexing method

## Abstract

On the basis of realizing regional navigation, the Quasi-Zenith Satellite System (QZSS) has advanced navigation function, which leads to the broadcasting of more signals in a single frequency of QZSS signals. Current signal transmission technology cannot solve this problem, so it is necessary to design a signal multiplexing method. The current QZSS satellite interface document does not disclose the multiplexing modulation method of the signal transmission, which has a certain impact on the acquisition of high-precision observation data and further data processing. The iGMAS (International GNSS Monitoring & Assessment System) Monitoring and Evaluation Center of the 54th Research Institute of China Electronics Technology Group Corporation has used the low-distortion data acquisition and processing platform and refined signal software receiving processing algorithm of the iGMAS to complete the signal acquisition and analysis of QZSS satellites. Analysis of the multiplexing and modulation method and signal characteristics for the QZSS has been carried out, which can provide a reference for the design and data processing of high-precision receivers.

## 1. Introduction

**Figure 1:**
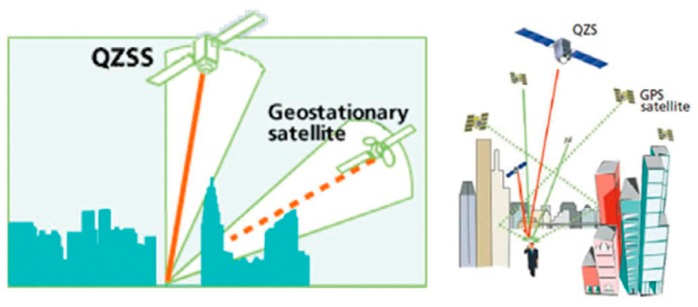
Seamless service of Quasi-Zenith Satellite System (QZSS) system and GPS system.

The Quasi-Zenith Satellite System (QZSS) was first proposed by the Japan Open Committee in June 2000. In its report “Improving the Open Space System of Japan and Expanding New Fields of Space Utilization,” the QZSS system was used as the key construction system for space infrastructure planning. The system started construction in 2002 [1]. The first satellite QZS-1 was launched in 2010, and three satellites QZS-2, QZS-3, and QZS-4 were launched in 2017. The QZSS system mainly solves the problem where the positioning services provided by the GPS system cannot meet the navigation and positioning needs of urban vehicle users in most urban canyons in Japan [2], as shown in Figure 1, by building a regional satellite navigation system with positioning, mobile communication, and broadcasting functions. It provides auxiliary enhanced functions for the GPS system and greatly improves the accuracy and availability of navigation in Japan [3].

In order to achieve the purpose of assisting GPS system navigation enhancement, QZSS satellite navigation signals were designed with compatibility and interoperability with GPS navigation signals at the beginning of the design. Therefore, the signal components broadcast by QZSS satellites include L1C/A, L1C, L2C, and L5 signals [4], which are consistent with GPS satellites, as shown in Table 1. It ensures that the QZSS system requires minimal changes to the user system technical requirements and the receiver design based on the GPS receiver. In addition to broadcasting signals compatible with the GPS system, two additional signals are broadcast. One is the L1-SAIF(Submeterclass Augmentation with Integrity Function) signal at 1575.42 MHz. The main purpose is to provide users in Japan with wide-area differential GPS corrections and integrity information, and to be compatible with space-based augmentation systems [5]. The other is the LEX(L-band Experiment) signal at 1287.75 MHz. The LEX signal itself is an experimental navigation signal with a higher navigation message rate. Its modulation method is code shift keying (CSK) [6].

Traditional navigation signals use QPSK’s multiplexing modulation method to complete the broadcast of the two signal components. However, as satellite navigation applications in the civilian and military fields continue to expand, the navigation system needs to be compatible with existing navigation signals while adding new navigation signals. Due to limited navigation frequency resources, more signals need to be modulated at the same frequency. However, the navigation load is a power-constrained system. Considering the non-linear characteristics of the power amplifier, the selection and combination of signal modulation and multiplexing methods will affect the power ratio and phase relationship in the respective signals’ broadcast process [7]. In the distribution of constellation points of different modulation methods, the corresponding signal amplitude of different signal constellation points will be affected by the nonlinearity of the satellite power amplifier [8]. Therefore, it is very important to choose a multiplexing modulation method for transmitting navigation signals reasonably, especially for QZSS L1. QZSS L1 contains at least four signals, which are L1C/A, L1C-pilot, L1C-data, and L1-SAIF. The modulation method and pseudo-code generation method of the QZSS satellite signal have been specified in the official ICD(Interface Control Document) file, but the multiplexing method and broadcasting method of its satellite transmission are not disclosed. Therefore, it is necessary to monitor its signal characteristics and analyze the multiplexing method to provide a reference for the design of the satellite signal broadcasting system of the next-generation navigation system.

The iGMAS Monitoring and Evaluation Center (Shijiazhuang) of State Key Laboratory of Satellite Navigation System and Equipment Technology of the 54th Research Institute of China Electronics Technology Group Corporation has completed the acquisition of QZSS satellite signals by using the central signal quality monitoring system 15 m large-scale parabolic antenna, and performed original signal layer detailed analysis, the corresponding analysis of its signal component composition, the multiplexing mode, and broadcast mode.

## 2. High-Precision Signal Quality Monitoring and Evaluation Technology

As the general navigation receiver mainly uses an omnidirectional low-gain antenna to complete the capture and tracking of the QZSS signal, and finally acquires the navigation messages and pseudoranges, it is impossible to analyze from the original layer of the QZSS signal. In addition, in the low-gain antenna scenario, the signal-to-noise ratio of the QZSS navigation signal received on the ground is −30 dBm, and the signal power is much smaller than the noise power, which will also seriously affect the accuracy of the analysis results of the QZSS satellite broadcast signals. Therefore, only high-gain large-diameter directional antennas can be used to complete the real-time acquisition and storage of signals. In addition, tools such as MATLAB can be used to complete the analysis of the stored data files. According to such a monitoring and evaluation process, high-precision signal quality monitoring and evaluation technology is mainly divided into two parts, a low-distortion data acquisition and processing technology and refined signal software receiving and processing technology.

The key point of low-distortion data acquisition and processing technology is how to obtain the original signal transmitted by the satellite in a low-distortion scene. The main solutions include the following three points. The first point is high-gain antenna reception, which minimizes effects of interference and noise on the signal. The second point is the use of low-distortion signal acquisition links, which simplifies the signal analog transmission link as much as possible by using the high sampling rate for direct RF acquisition. The third point is to calibrate the channel characteristics of the transmission link and then perform the distortion recovery on the digital terminal of the signal.

**Figure 2:**
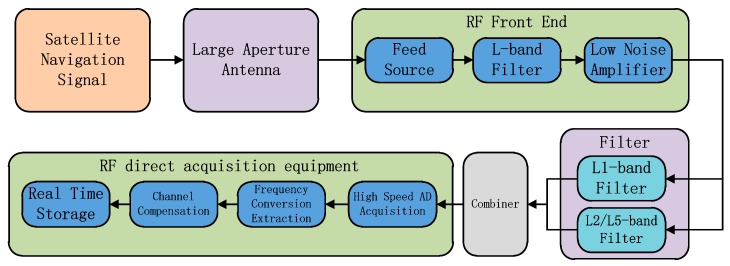
Low-distortion data acquisition and processing technology.

The basic flow of the low-distortion data acquisition and processing technology is shown in Figure 2. The high-gain large-diameter antenna is used to complete the real-time stable tracking of space navigation satellites, which are above 30° elevation. The navigation satellite signals pass through the feed, L-band filter, and low-noise amplifier in order. The RF front-end filter bandwidth covers 1100–1700 MHz. The main function of the filter is to filter out the impact of other frequency bands on the navigation frequency band at the analog end and finally use the low-noise amplifier to complete signal amplification. Then, the crossover filter is used to complete the filtering process of the observation frequency band. The purpose is to filter out the interference that may be introduced in the non-navigation band in the L band and avoid aliasing during direct RF acquisition [9]. Finally, after the multiple signals are combined, the acquisition is completed by the RF direct acquisition equipment. Inside the RF direct acquisition equipment, in addition to the sampling by high-speed AD(Analogue-to-Digital), digital frequency conversion extraction processing is performed on each frequency point on the FPGA(Field Programmable Gate Array) to reduce the sampling rate of stored data, thereby improving the efficiency of data analysis. The AD signal sampling rate is 1 GHz and the digital samples are 14 bits. Channel compensation is performed on the extracted signal [10]. The parameters of the compensation filter are determined according to the calibration results of the entire acquisition channel to ensure that the acquisition signal is close to no distortion. The general calibration method broadcasts the sweep signal of the observation frequency band at the wired entrance of the antenna feed, and uses the acquisition equipment to complete the acquisition of the sweep signal. The calibration results of the acquisition channel can be obtained by using the multi-frequency sweep signal’s amplitude–frequency variation characteristics at different frequencies. The FIR filter coefficients are designed to complete the channel compensation and reflect it in the compensation filter to ensure that the in-band flatness without distortion of the acquired signal approaches ± 0.2 dB/MHz. Finally, the high-speed bus is used to complete the real-time data storage.

The key point of the refined signal software receiving and processing technology is how to finely analyze the acquired navigation satellite signals. The main solutions include the following two points. The first point is the high-precision signal processing. It retains as many sampling signal sampling bits as possible. A local data carrier with multiple data bits and local pseudo-code with a high sampling rate are used to perform operations on the collected data. The second point is to accurately obtain the zero-IF(Intermediate Frequency) signal of each integration cycle and use a low-dynamic-range tracking loop to track each Doppler and code phase of each integrated periodic signal, which are eliminated according to the measurement results.

**Figure 3:**
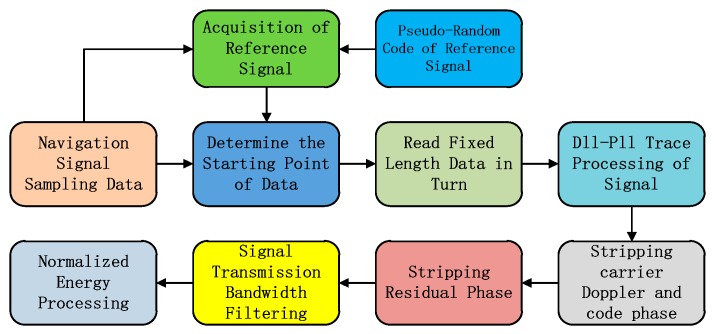
Refined signal software receiving and processing technology.

The basic flow of the refined signal software receiving and processing technology is shown in Figure 3. The collected signal data are used for the capture of civil reference signals. The starting point of reading the data is determined. The original signal of the integration cycle length is sequentially read according to the starting point. The tracking processing of the read signal is completed by using the DLL(Delay Locked Loop) and PLL(Phase Locked Loop) tracking loop. An accurate carrier Doppler and code phase can be obtained [11]. The observation results of the tracking loop are used to obtain the zero-IF signal with the stripped Doppler residual code phase. Residual code phase stripping is performed on the zero-IF signal. The transmission bandwidths are filtered on the observed frequency points. Finally, the energy normalization process is performed to obtain the final refined signal quality test signal [12]. This signal is used to complete the processing in the corresponding frequency domain spectrum, modulation domain constellation, and correlation domain correlation peaks.

## 3. Monitoring and Analysis of QZSS Satellite Signal Characteristics

After the full-band acquisition of QZSS satellite signals, the L1, L2, L5, and L6 frequency points can be separated, and the characteristics of the original layer of the signal can be extracted and analyzed in turn. The analysis can be divided into a single-frequency point overall analysis and a single-component analysis. The single-frequency point analysis mainly includes the characteristics analysis of the frequency domain and the modulation domain. The modulation domain is mainly used to characterize the two-dimensional time domain characteristics of the signal IQ(In-phase/Quadrature) branch [13]. The single-component analysis includes the correlation domain and measurement domain characteristics analysis, which mainly include the correlation peak of each signal component and the normalized power ratio within the emission bandwidth.

### 3.1. Monitoring and Analysis of L1 Frequency Point Signal Characteristic

**Figure 4:**
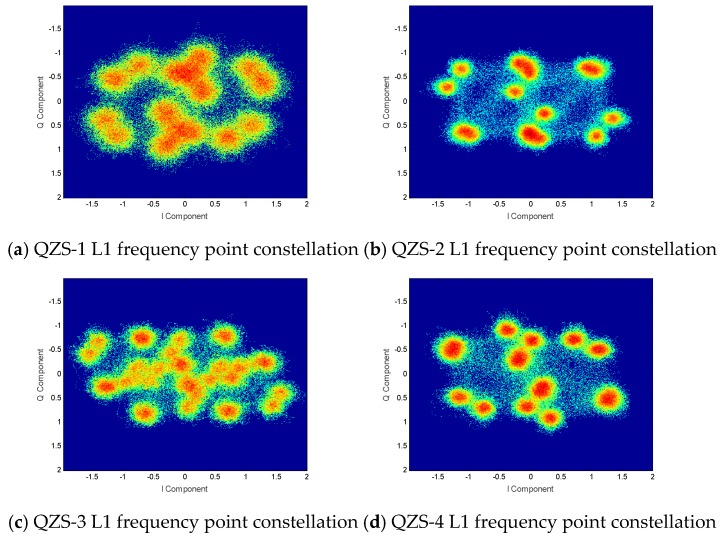
QZSS satellite L1 frequency constellations.

Through the overall analysis of the L1 frequency signal, the constellation diagram of four QZSS satellites is shown in Figure 4. The elevation angle of the QZS-1 satellite in the experiment is 37.2°. The elevation angle of the QZS-2 satellite in the experiment is 66.8°. The elevation angle of the QZS-3 satellite in the experiment is 44.0°. The elevation angle of the QZS-4 satellite in the experiment is 55.2°.

It can be clearly seen from the figure that the distributions of the constellation diagrams of the four satellites are significantly different. The QZS-3 satellite has 32 constellation points, and the other three satellites have 16 constellation points. As the signal components used by the QZSS are all by the non-return to zero (NRZ) coding method [14], one signal component represents two constellation points, two signal components form 22 = 4 constellation points, and so on. It can be inferred that QZS-3 has five signal components, and the remaining satellites have four signal components.

**Figure 5:**
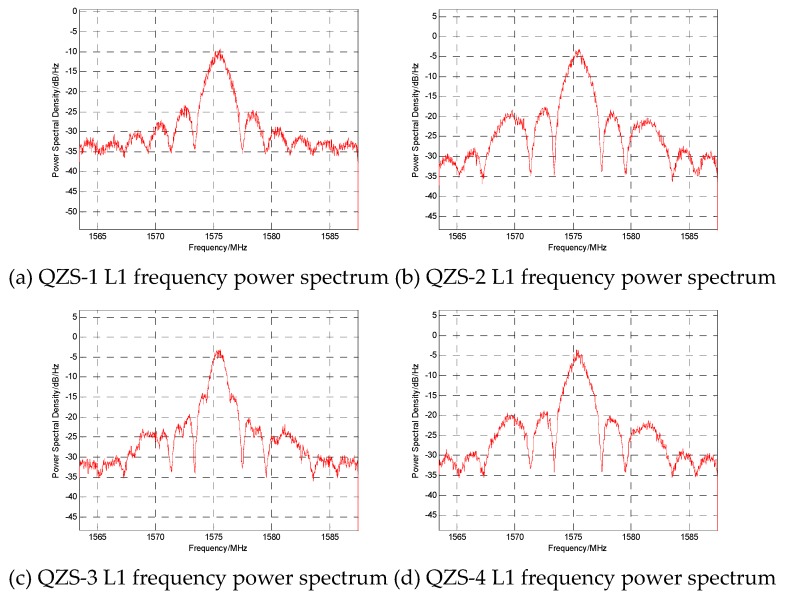
QZSS satellite L1 frequency point power spectra.

The L1 frequency power spectra of four QZSS satellites are shown in Figure 5. It can be seen from the figure that the main lobe of the signal of the QZS-3 satellite L1 signal is significantly different from those of the other satellites. The initial guess is that it is caused by five signal components of the QZS-3 satellite. In addition, the second lobe of the signal of the QZS-1 satellite is different from those of the QZS-2 and QZS-4 satellites. This may be caused by the different modulation methods used for the signal components inside.

A detailed analysis of the signal components of the four satellites at the L1 frequency point shows that there are indeed five signal components inside QZS-3. As shown in Table 2, the QZS-3 satellite transmits two L1-SAIF signals. That is why there are 32 points in the constellation diagram. In addition, the extra L1-SAIF signal power accounted for 46.67%, accounting for the main signal energy. The main reason is that QZS-3 belongs to the geosynchronous orbit satellite (GEO), and it provides Satellite-Based Advanced services with the help of the L1-SAIF signal. For GPS-compatible L1 C/A, L1Cp, and L1Cd signals, the pseudo-code broadcast by each satellite is fully completed according to the ICD, with only a slight difference in transmit power. However, on the QZS-1 satellite, the configuration of L1Cd and L1Cp is out of phase. Therefore, the modulation of each signal component is further studied.

**Figure 6:**
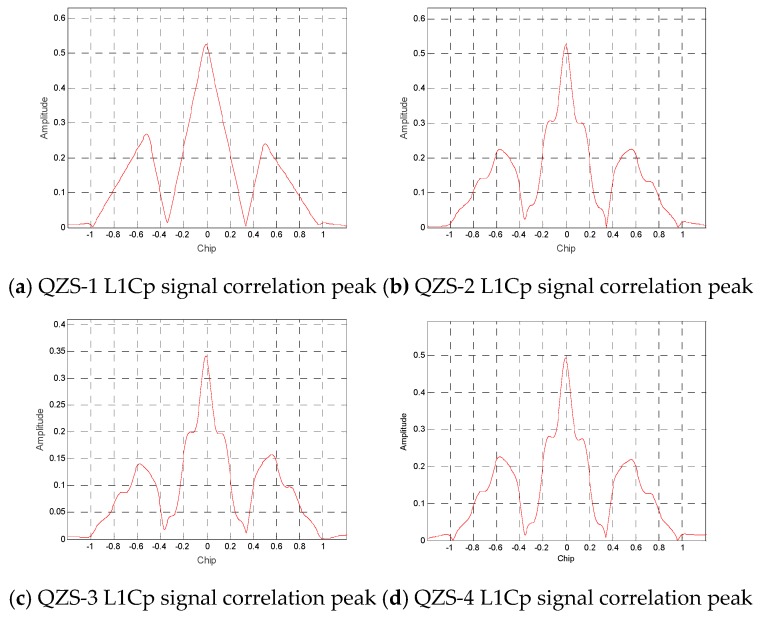
QZSS satellite L1Cp correlation peak.

Through the study of the correlation peaks, as shown in Figure 6, at the L1C frequency point, only the L1Cp signal component of the QZS-1 satellite uses BOC(Binary-Offset-Carrier) (1,1), and the other satellites use the TMBOC(Time-Multiplexed Binary-Offset-Carrier) (6,1,4/33) modulation method. The L1Cp signal of the QZS-1 satellite still uses BOC (1,1) modulation, which explains why different envelopes are generated on the side lobes of the power spectrum. The QZS-1 satellite was launched in 2010. The earlier launch year resulted in a lack of consistency with the latest GPS interface files. In addition, the phase angle between the L1-SAIF signal and other signal components obviously has no regularity. It can be clearly inferred that the L1-SAIF signal is transmitted using a separate channel and antenna.

### 3.2. Monitoring and Analysis of L2 Frequency Point Signal Characteristic

**Figure 7:**
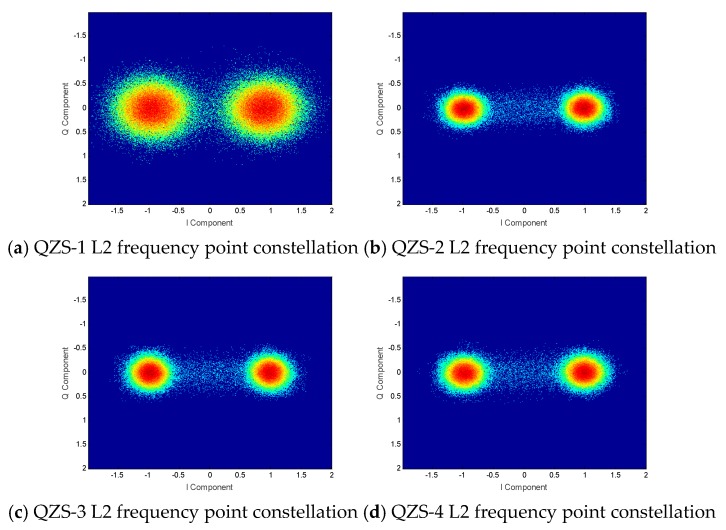
QZSS satellite L2 frequency constellations.

The constellation diagram and power spectra of the L2 frequency of the QZSS satellite are shown in Figure 7. It can be seen from the figure that the signal of the L2 frequency is completely consistent with the L2 signal system designed by GPS. It uses the BPSK(Binary Phase Shift Keying) (1) modulation method, so there is no need for further analytical research.

### 3.3. Monitoring and Analysis of L5 Frequency Point Signal Characteristic

**Figure 8:**
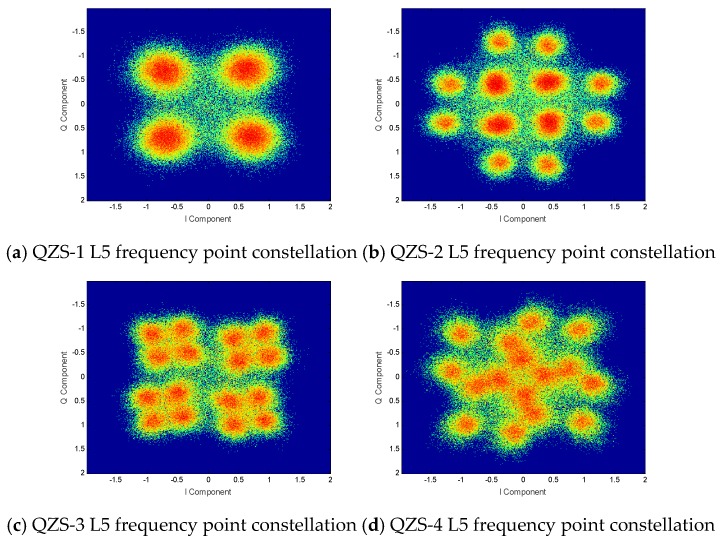
QZSS satellite L5 frequency constellations.

For the overall analysis of the L5 frequency signal, the constellation diagrams of four QZSS satellites are shown in Figure 8. It can be clearly seen from the figure that the QZS-1 satellite has only four constellation points, which belongs to the QPSK multiplex modulation method. The constellation diagrams of the other three satellites have 16 constellation points, which means that the signal has four signal components. Those three constellation diagrams are also significantly different. Similar to the L1 frequency point constellation diagram, the reason for this result is that there is a single-channel transmission of some signal components.

By analyzing the internal characteristics of the signal components, the analysis results are shown in Table 3. It can be found that except for the QZS-1 satellite, the other three satellites have transmitted the other pseudo-code L5 frequency QPSK signals with lower power. The direct phase relationship of the main component L5 signal has no regularity. The preliminary analysis is due to a separate channel to complete the signal transmission.

### 3.4. Monitoring and Analysis of L6 Frequency Point Signal Characteristic

**Figure 9:**
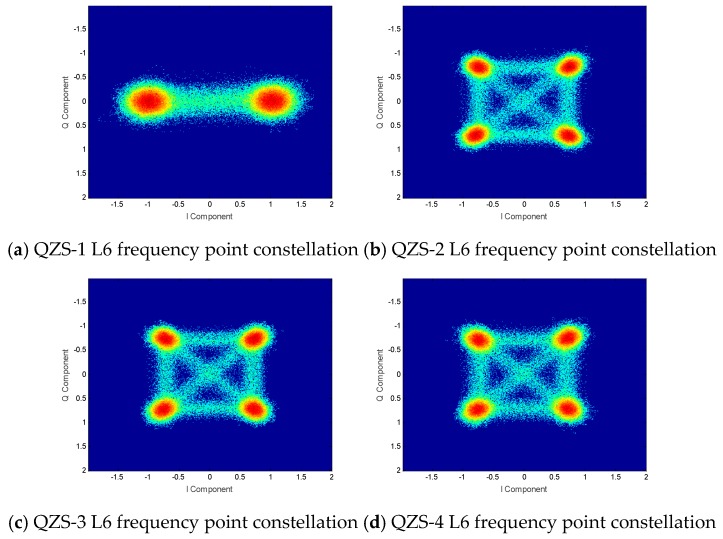
QZSS satellite L6 frequency constellations.

The constellation diagram and power spectra of the QZSS satellite L6 frequency are shown in Figure 9. From the figure, it can be seen that the signal at the L6 frequency is completely consistent with the signal system specified by ICD, which means that QZS-1 has only one signal component, and the other three satellites have two orthogonal signal components [13].

## 4. Analysis of QZSS Satellite Signal Multiplexing Method

### 4.1. Analysis of L5 Frequency Point Signal Multiplexing Method

**Figure 10:**
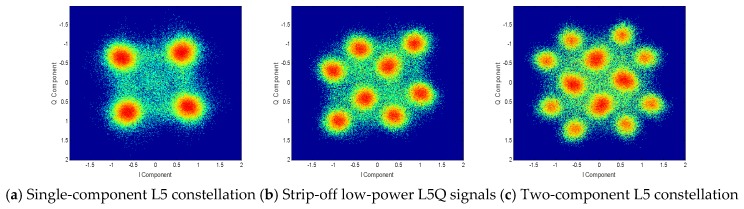
QZS-2 satellite L5 frequency signal constellation diagram.

First, the characteristics of the broadcast signal at the L5 frequency point are studied. Taking QZS-2 as an example, the low-power L5 signal in the baseband signal is recovered and removed. The single-component L5 signal constellation can be obtained, as shown in Figure 10a. It can be obtained that the expression of the L5 frequency baseband signal broadcast at this time is

(1) $$S_{L5\_D}\left(t\right)=\sqrt{P_{I}}D_{I}\left(t\right)C_{I}\left(t\right)+j\sqrt{P_{Q}}D_{Q}\left(t\right)C_{Q}\left(t\right).$$

In the above formula, $P_{I}$ and $P_{Q}$ are the power of the signals of the two branches I and Q, respectively. $D_{I}\left(t\right)$ and $D_{Q}\left(t\right)$ are the subcodes or messages of the L5I code signal and L5Q code signal, respectively. $C_{I}\left(t\right)$ and $C_{Q}\left(t\right)$ are the pseudo-codes of the L5I and L5Q signals, respectively. As can be seen from Figure 10b, the original QPSK signal has a displacement in a certain vector direction. The displacement direction depends on the input sign of the low-power L5I. Suppose the moving vector is w=A+j×B, and |w|=1. When the multiplication of the message and chip of the low-power L5I signal is 1, the $S_{L5\_D}\left(t\right)$ vector is added by w. When the multiplication of the message and chip is −1, the $S_{L5\_D}\left(t\right)$ vector is reduced by w. Thus, the signal expression in Figure 10b can be written as

(2) $$\begin{array}{l} S_{L5\_T}\left(t\right)=S_{L5\_D}\left(t\right)+\sqrt{P_{I2}}\\ \;\;\;\;\;\;\;\cdot \left[\left(D_{I2}\left(t\right)C_{I2}\left(t\right)\cdot 0.5+0.5\right)\cdot w+\left(-D_{I2}\left(t\right)C_{I2}\left(t\right)\cdot 0.5+0.5\right)\cdot \left(-w\right)\right]. \end{array}$$

Sorting out the above formula gives

(3) $$\begin{array}{l} S_{L5\_T}\left(t\right)=\sqrt{P_{I}}D_{I}\left(t\right)C_{I}\left(t\right)+j\sqrt{P_{Q}}D_{Q}\left(t\right)C_{Q}\left(t\right)\\ \;\;\;\;\;\;+\sqrt{P_{I2}}D_{I2}\left(t\right)C_{I2}\left(t\right)\cdot w. \end{array}$$

Let $w=e^{j\theta }$, $\theta =\arctan\left(\frac{A}{B}\right)$. The above formula can be rewritten as

(4) $$S_{L5\_T}\left(t\right)=\sqrt{P_{I}}D_{I}\left(t\right)C_{I}\left(t\right)+j\sqrt{P_{Q}}D_{Q}\left(t\right)C_{Q}\left(t\right)+\sqrt{P_{I2}}D_{I2}\left(t\right)C_{I2}\left(t\right)\cdot e^{j\theta }.$$

It can be seen from the above formula that the main components of the L5I and L5Q signals are strictly modulated in accordance with the 90° phase, and that the low-power L5I signal and the other two signals can be separated on the baseband. The L5Q signal is added in the same way, as shown in Figure 10c. Suppose the added L5Q vector signal is v=C+j×D, and |v|=1. Considering that vector w is strictly perpendicular to vector v, v can be rewritten as v=B−j×A, and |v|=1. The exponential form is $w=e^{j\beta }$, where $\beta =\arctan\left(\frac{B}{-A}\right)=\theta +\frac{\pi }{2}$. The signal broadcast by the L5 frequency point of the QZSS satellite is completely expressed as

(5) $$S_{L5}\left(t\right)=\sqrt{P_{I}}D_{I}\left(t\right)C_{I}\left(t\right)+j\sqrt{P_{Q}}D_{Q}\left(t\right)C_{Q}\left(t\right)+\sqrt{P_{I2}}D_{I2}\left(t\right)C_{I2}\left(t\right)\cdot e^{j\theta }+\sqrt{P_{Q2}}D_{Q2}\left(t\right)C_{Q2}\left(t\right)\cdot e^{j\left(\theta +\frac{\pi }{2}\right)}.$$

Sorting out the above formula gives

(6) $$S_{L5}\left(t\right)=\sqrt{P_{I}}D_{I}\left(t\right)C_{I}\left(t\right)+j\sqrt{P_{Q}}D_{Q}\left(t\right)C_{Q}\left(t\right)+e^{j\theta }\left(\sqrt{P_{I2}}D_{I2}\left(t\right)C_{I2}\left(t\right)+j\sqrt{P_{Q2}}D_{Q2}\left(t\right)C_{Q2}\left(t\right)\right).$$

Modulating the signal to the frequency band:

(7) $$S_{L5\_R}\left(t\right)=\sqrt{P_{I}}D_{I}\left(t\right)C_{I}\left(t\right)\cos\left(2{\pi f}_{c}t+\theta_{0}\right)+j\sqrt{P_{Q}}D_{Q}\left(t\right)C_{Q}\left(t\right)\sin\left(2{\pi f}_{c}t+\theta_{0}\right)\;+e^{j\theta }\left(\sqrt{P_{I2}}D_{I2}\left(t\right)C_{I2}\left(t\right)\cos\left(2{\pi f}_{c}t+\theta_{0}\right)+j\sqrt{P_{Q2}}D_{Q2}\left(t\right)C_{Q2}\left(t\right)\sin\left(2{\pi f}_{c}t+\theta_{0}\right)\right).$$

According to the above derivation, it can be concluded that the QZSS satellite L5 signal uses two independent channels to transmit. The advantage is to ensure that the two signals are transmitted using a constant envelope transmission system to reduce the impact of the nonlinearity of the power amplifier and avoid the distortion of the signal caused by the constant envelope modulation using too many signal component paths. In addition, broadcasting additional L5 frequency signals can increase the number of available satellites for ground users and improve the availability of navigation [14].

### 4.2. Analysis of L1 Frequency Point Signal Multiplexing Method

**Figure 11:**
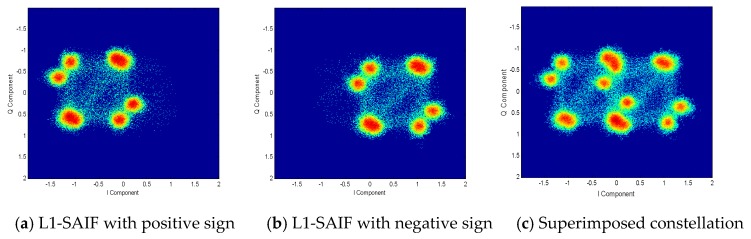
QZS-2 satellite L1 frequency point signal constellation.

According to the analysis above, the L1-SAIF signal is transmitted in a separate channel. Therefore, by statistically distinguishing the sign of the pseudo-code of the L1-SAIF signal and the sign of the message, the constellation diagram can be obtained, as shown in Figure 11 (take the QZS-2 satellite as an example).

According to the derivation results in the previous section, the expression of the L1 frequency point signal can be written as

(8) $$S_{L1}\left(t\right)=\sqrt{P_{G}}\mathrm{Multiplex}(S_{\mathrm{CA}}\left(t\right),S_{L1\mathrm{Cp}}\left(t\right),S_{L1\mathrm{Cd}}\left(t\right))+\sqrt{P_{\mathrm{SAIF}}}D_{SAIF}\left(t\right)C_{\mathrm{SAIF}}\left(t\right)\cdot e^{j\theta }.$$

In the above formula, Multiplex($S_{\mathrm{CA}}\left(t\right),S_{L1\mathrm{Cp}}\left(t\right),S_{L1\mathrm{Cd}}\left(t\right)$) is the multiplexed and modulated GPS system compatible signals. $P_{G}$ and $P_{\mathrm{SAIF}}$ are the power of compatible signals and SAIF signals, respectively. $D_{SAIF}\left(t\right)$ and $C_{\mathrm{SAIF}}\left(t\right)$ are the messages and pseudo-codes of the SAIF signals, respectively. θ is the carrier phase deviation between the SAIF signal and the compatible signal.

Similarly, for the QZS-3 satellite L1 frequency signal with two SAIF signals, the expression can be written as

(9) $$\begin{array}{l} S_{L1}\left(t\right)=\sqrt{P_{G}}Multi(S_{CA}\left(t\right),S_{L1Cp}\left(t\right),S_{L1Cd}\left(t\right))+\sqrt{P_{SAIF1}}D_{SAIF1}\left(t\right)C_{SAIF1}\left(t\right)\cdot e^{j\theta }+\\ \;\;\;\;\;\;\;\;\;\;\sqrt{P_{SAIF2}}D_{SAIF2}\left(t\right)C_{SAIF2}\left(t\right)\cdot e^{j\beta }. \end{array}$$

In the above formula, θ and β are carrier phase deviations between two SAIF signals and compatible signals.

**Figure 12:**
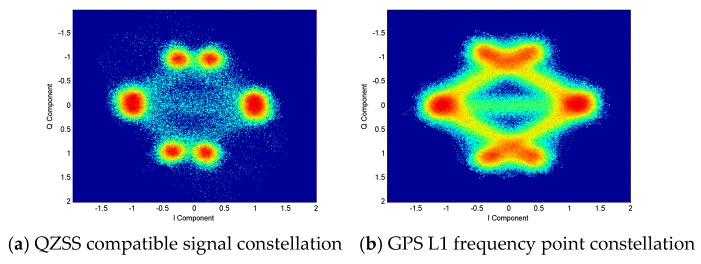
Comparison of QZSS L1 frequency compatible signal and GPS signal constellation.

For C/A, L1Cp, and L1Cd multiplexed signals, the constellation diagram can be observed, as shown in Figure 12. Comparing with the GPS signal constellation, because the phase relationship of the three signals is in-phase or orthogonal, it can be speculated that these three signals are using coherent adaptive subcarrier modulation (CASM) constant envelope modulation [15]. The only difference is that QZSS compatible signals are internally M code signals. As M code signals use BOC (10,5) modulation [16], the baseband signal will generate more high-frequency components. Therefore, there will be more distortion after filtering, and the QZSS satellite constellation points are relatively clear.

The CASM multiplexing modulation method used by QZSS satellites places the C/A signal on the Q branch and places the L1Cp and L1Cd signals on the I branch. The synthesized mathematical expression is

(10) $$\begin{array}{l} S_{G}\left(t\right)=\sqrt{P_{G}}Multi\left(S_{CA}\left(t\right),S_{L1Cp}\left(t\right),S_{L1Cd}\left(t\right)\right)=\sqrt{P_{CP}}S_{L1\mathrm{Cp}}\left(t\right)+\sqrt{P_{CD}}S_{L1\mathrm{Cd}}\left(t\right)+\\ \;\;\;\;\;\;\;\;j(\sqrt{P_{CA}}S_{\mathrm{CA}}\left(t\right)-\sqrt{P_{M}}S_{\mathrm{CA}}\left(t\right)S_{L1\mathrm{Cp}}\left(t\right)S_{L1\mathrm{Cd}}\left(t\right)). \end{array}$$

In the above formula, $P_{CP}$, $P_{CD}$, $P_{CA}$, and $P_{M}$ are the power of L1Cp, L1Cd, C/A, and intermodulation components, respectively. Through the adjustment of the intermodulation power, the constellation points of the multiplexed signal are distributed on the unit circle. In addition, for QZS-1 compatible signals, L1Cp is placed on the Q branch.

In summary, the compatible part of the QZSS L1 signal with GPS uses CASM constant envelope modulation to ensure a constant signal output envelope, which greatly reduces the impact of the non-linearity of the power amplifier and improves the quality of navigation signals. For L1-SAIF signals, a separate transmission channel is used, which is convenient for the separate control of wide-area differential signals.

## 5. Conclusions

In this paper, through the in-depth monitoring and analysis of the QZSS signal system, identification of the broadcast mode and modulation and multiplexing mode of all frequency points of the QZSS signal is completed. The following conclusions can be drawn:

- At the L2 frequency, QZSS satellites broadcast messages completely in accordance with the BPSK multiplex modulation method of the GPS L2 frequency civil signals. At the L6 frequency, the QZSS satellites broadcast messages in accordance with the requirements of ICD by implementing a simple CSK modulation and QPSK multiplexing.
- QZSS satellites have at least two channel signal transmission capabilities at the L5 frequency point. The two channels can be used independently to transmit. The broadcasting method of each single channel is consistent with the ICD regulations, but the broadcasting method of two channels is not disclosed. The advantage is that it can provide more available satellites for regional navigation coverage, and improve the accuracy and availability of navigation services.
- QZSS satellites use a separate channel to transmit L1-SAIF signals. In addition, the QZS-3 satellite, as a GEO satellite, separately transmits two channels of L1-SAIF signals to adapt to different differential positioning enhancement scenarios.
- The GPS system compatible signals of the QZSS satellite L1 frequency point only broadcast the C/A signal and L1C signal. The L1C signal of the QZS-1 satellite adopts the BOC (1,1) old system modulation method. The multiplexing method is the CASM multiplexing method by placing the L1Cp signal on the Q branch and placing the C/A and L1Cd signals on the I branch. For the other three satellites, the L1C signal adopts TMBOC (6,1,4/33) modulation (standard requirements). The multiplexing method is the CASM multiplexing method by placing the C/A signal on the Q branch and placing the L1Cp and L1Cd signals on the I branch. This multiplexing method is consistent with the multiplexing method used by GPS satellites and has not been publicly released in ICD. As the high-order modulation signal is not broadcast, the distortion of the filtered signal is less, which ensures the high-precision scene application of the navigation signal.

## Figures and Tables

**Table 1:** QZSS system broadcast signals and modulation methods.

Signal Frequency Point	Frequency (MHz)	Signal Components	Modulation Method	Signal Bandwidth (MHz)	Compatibility
L1	1575.42	L1 C/A	BPSK(1)	24	Compatible with GPS L1 signals
		L1C pilot	TMBOC		
		L1C data	BOC(1,1)		Similar to SBAS signal
		L1-SAIF	BPSK(1)		
L2	1227.6	L2CM	BPSK(1)	24	Compatible with GPS L2 signals
		L2CL	BPSK(1)		
L5	1176.45	L5I	BPSK(10)	25	Compatible with GPS L5 signals
		L5Q	BPSK(10)		
L6(LEX)	1278.75	L6I	CSK	42	Experimental signal
		L6Q	CSK		

**Table 2:** QZSS satellite L1 frequency signals composition analysis.

Satellite Number	QZS-1	QZS-2	QZS-3	QZS-4
L1C/A PRN	PRN 193	PRN 194	PRN 199	PRN 195
L1C PRN	PRN 193	PRN 194	PRN 199	PRN 195
L1-SAIF PRN	PRN 183	PRN 184	PRN 187	PRN 185
			PRN 189	
L1C/A power ratio	25.21%	17.63%	9.82%	20.38%
L1C pilot power ratio	27.62%	27.73%	11.68%	24.37%
L1C data power ratio	9.04%	11.05%	3.96%	7.32%
L1-SAIF power ratio	23.09%	29.5%	46.67%	29.93%
			17.6%	
L1C/A phase angle	0°	0°	0°	0°
L1C pilot phase angle	90°	90°	90°	90°
L1C data phase angle	0°	90°	90°	90°
L1-SAIF phase angle	58°	27°	46°	55°
			6°	

**Table 3:** QZSS satellite L5 frequency signals composition analysis.

Satellite Number	QZS-1	QZS-1	QZS-3	QZS-4
PRN	PRN 193	PRN 194 (principal components) PRN 196	PRN 199 (principal components) PRN 197	PRN 195 (principal components) PRN 200
L5I power ratio (principal components)	44.13%	32.36%	39.84%	30.33%
L5Q power ratio (principal components)	45.18%	31.60%	39.41%	29.85%
L5I power ratio (other components)	N/A	13.72%	6.31%	14.80%
L5Q power ratio (other components)	N/A	14.41%	6.02%	14.64%
L5I phase (principal components)	0	0	0	0
L5Q phase (principal components)	90	90	90	90
L5I phase (other components)	N/A	45	4	73
L5Q phase (other components)	N/A	135	94	163
